# A Case of Amaurosis, *from a Gun-Shot Wound*

**Published:** 1839-12-21

**Authors:** John B. Colhoun

**Affiliations:** Philadelphia


### A CASE OF AMAUROSIS,

*From a gun-shot wound.*

BY JOHN B. COLHOUN, M. D.

In July, 1838, I was called to visit a negro man, belonging to William Pickett, Esq., Yazoo County, Mississippi, who had been shot, at the distance of about seventy yards, with a double-barrelled gun, both barrels of which were discharged at him, when in the act of pilfering on an adjoining plantation. The piece had been charged for deer shooting, and contained several medium sized buck shot surmounted by an ounce ball, in each barrel.

Upon examination, ten hours after the wound had been inflicted, I found that a large ball had entered the back, at a very oblique angle, over the dorsum of the left scapula, near its spine, and, taking the course of the ribs, had passed round through the axilla and lodged in the mammary region, somewhat below its point of entry. The scapula appeared to be extensively fractured, several spicula of which were found at the orifice of the wound. Owing to the great distension of the parts, from ecchymosis, I could not, at this time, accurately ascertain the position of the ball. I now observed his eyes, particularly the right eye, to be inflamed, without any apparent external violence having been done either to them or the adjacent parts. The patient now informed me that, simultaneously with the discharge of the gun, his vision had become confused and indistinct, and so gradually increased, until, in a short time, so far as surrounding objects were discernible, it was completely gone. On closer inspection, the pupils presented a nebulous appearance, were much dilated, and (owing to the angular shape of the irides, which, under the strongest light, remained flaccid and immoveable,) extremely irregular in their form; the corneæ, too, had lost much of their usual brilliancy, and were of a dull glassy hue, which seemed, in a measure, to be unconnected with the cloudiness existing in the chambers of the eye. The retina, notwithstanding the condition of the iris, still so far retained its susceptibility as to enable the patient to discover day from night, or, when in a darkened room, to detect the intervention of a person, or other dense object, between him and a strong current of light; this, as frequently occurs in amaurosis, was more apparent to him from an oblique than a direct glance. In other respects the functions of both eyes were completely lost; the organization, however, of the right eye, to all appearance, had been more extensively injured than that of the left. The wound already described was the only apparent one he received, not the slightest contusion, or even abrasion, being discoverable elsewhere; nor is he conscious of having received any blow or other violence on or about the eyes. In three or four days, the ecchymosis being sufficiently reduced, I extracted the ball; adhering to it, were several spicula of bone, which had been forced throughout the course of the wound. The ball was not flattened, but, from its contact with the scapula, so cut and indented as to have entirely lost its original surface. No constitutional symptoms of moment intervened, and the wound speedily healed without any unpleasant symptoms. A slight ulcerated speck appeared on the fourth day, at the extreme outer edge of the right cornea, which being removed by a single application of lunar caustic, the ophthalmia readily yielded to the usual general and topical treatment.

Until January of the present year, I have seen the case almost weekly, during which time, the nebulary appearance of the aqueous humour was gradually converted into semi-opacity; and, *pari passu* with its increase, the sensibility of the retina diminished, until, in a few months, it became wholly extinct. I have frequently endeavoured, by suddenly presenting a strong light, to induce a contraction of the iris, but invariably without effect. The nebula appeared to exist in the anterior chamber, though, probably, it may have extended to the vitreous humour. A month since, I learned from a medical friend, who kindly saw the case at my request, that no change has since intervened, the patient remaining hopelessly blind.

This case, so far as my information extends, is anomalous, and, even admitting instantaneous amaurosis in the left eye to have resulted from sympathy with the right, exceedingly difficult to explain. The testimony of the patient is conclusive as to both eyes being simultaneously and immediately affected. Should we, however, reject his statement, (which, as he could have no object in deceiving, we should not,) still, blindness must have ensued in ten hours, at furthest, after the discharge of the gun. In either attitude the case is sufficiently remarkable.

With the head in its natural position, the same discharge that would strike the scapulary region, at a very oblique angle, would likewise range obliquely with the right eye, and, as in analogous cases of gun-shot wounds of other parts, might wound it, destroying its functions, without any external indication of violence being apparent. That this did occur is inferrential from the inflammation and other morbid appearances, *ab initio*, and, during the whole progress of the case, being more distinct in the right eye. This view of the matter, inferring as it does the immediate implication of the left eye by sympathetic action, is liable to the following objection. Cases of instantaneous amaurosis have always been attributable to causes acting equally and simultaneously upon both eyes; where one eye has been destroyed by sudden and direct violence, the loss of its fellow has been involved as a remote effect, and not as an immediate consequence; and, lastly, that violence was sustained at all, in this case, is, at best, only presumptive.

Fear, it has been suggested to me by a distinguished source, may have caused the amaurosis; this is possible—fear being an agent capable of equally affecting both eyes; Richter, Beer, Scarpa, and other eminent authorities on the subject, however, mention it only as a remote though frequent cause of amaurotic blindness; its exclusive agency in the present case, seems to be disproved by the difference of the morbid phenomena existing in the two eyes, and some other collateral circumstances. Fear and direct violence to the right eye may, probably, have co-operated in causing it.

Other possible constructions have, from time to time, suggested themselves to me, but which, as they are not, in view of all the features of the case, preferable to those already proposed, I shall not urge. I yield the case for publication as one of much interest in itself, in the hope, too, that it may receive from some other source a more satisfactory interpretation than I feel myself competent to give it.

*Philadelphia, Dec.* 8, 1839.

